# Eryptotic Phenotype in Chronic Myeloid Leukemia: Contribution of Neutrophilic Cathepsin G

**DOI:** 10.1155/2012/659303

**Published:** 2012-03-14

**Authors:** Rukmini Govekar, Poonam Kawle, Renjan Thomas, Suresh Advani, Sheena PV, Surekha Zingde

**Affiliations:** ^1^Advanced Centre for Treatment, Research and Education in Cancer, Tata Memorial Centre, Kharghar, Navi Mumbai 410 210, India; ^2^Department of Medical Microbiology, Faculty of Medicine and Health Sciences, Universiti Putra Malaysia, Serdang 43400, Malaysia; ^3^Department of Medical Oncology, Jaslok Hospital and Research Centre, Mumbai 400 026, India

## Abstract

In pathological conditions with concurrent neutrophilia, modifications of erythrocyte membrane proteins are reported. In chronic myeloid leukemia (CML), a myeloproliferative disease wherein neutrophilia is accompanied by enhanced erythrophagocytosis, we report for the first time excessive cleavage of erythrocyte band 3. Distinct fragments of band 3 serve as senescent cell antigens leading to erythrophagocytosis. Using immunoproteomics, we report the identification of immunogenic 43 kDa fragment of band 3 in 68% of CML samples compared to their detection in only 38% of healthy individuals. Thus, excessive fragmentation of band 3 in CML, detected in our study, corroborated with the eryptotic phenotype. We demonstrate the role of neutrophilic cathepsin G, detected as an immunogen on erythrocyte membrane, in band 3 cleavage. Cathepsin G from serum adsorbs to the erythrocyte membrane to mediate cleavage of band 3 and therefore contribute to the eryptotic phenotype in CML.

## 1. Introduction

Neutrophils are inflammatory cells which contribute to tissue repair. Antithetically, neutrophilia and associated effects of the neutrophilic proteases and oxidases can lead to additional pathology at the site of inflammation [1]. In patients with cardiovascular disease [2], ischemic stroke [3], and in pregnancy [4], neutrophilia co-occurs with modifications of erythrocyte membrane protein band 3, which is either aggregated or cleaved. The modified band 3 is a senescent cell antigen, which can mediate immune recognition and erythrophagocytosis [5, 6].

Neutrophilic proteases, mainly elastase, have been shown to cleave erythrocyte membrane proteins. Santos-Silva et al. [7] have demonstrated that cellular aging and incubation either with activated neutrophils or with neutrophilic elastase lead to aggregation of band 3 which they suggest could be due to cross-linking of proteolytic fragments [3]. In an *in vitro* study, neutrophilic elastase and cathepsin G are reported to degrade glycophorins on erythrocyte membrane [8]. The same group has shown degradation of glycophorins on the surface of erythrocytes from patients with myeloproliferative disease and attributed it to elastase [9]. Our study for the first time demonstrates the role of cathepsin G in the cleavage of erythrocyte band 3 in chronic myeloid leukemia (CML).

CML is a progressive myeloproliferative condition marked by neutrophilia, and patients also suffer from moderate to mild anemia. Altered profile of membrane-skeletal proteins in CML erythrocytes [10] leads to the expression of aggregated band 3 on cell surface and in turn enhances erythrophagocytosis *in vitro* [11]. In contrary to the reported aggregation [11], we observed excessively cleaved band 3 in majority of CML samples. Since distinct fragments of band 3 are recognized by serum IgG and thus induce erythrophagocytosis [12], we used immunoproteomics approach to identify the immunogens recognized by serum IgG in the membranes of CML erythrocytes. Fragments of band 3 as well as cathepsin G, which is a neutrophilic serine protease [13], were detected as antigens in the membranes of mainly CML erythrocytes. The *in vitro* studies to confirm the causal role of membrane-bound cathepsin G in proteolysis of band 3 is described.

## 2. Materials and Methods

### 2.1. Chemicals

Pepstatin A (P 4265), phenyl methyl sulfonyl fluoride (PMSF) (P 7626), sequencing grade trypsin (T 6567), and anti-band 3 N-terminus monoclonal antibody (B 9277) were purchased from Sigma-Aldrich Inc, USA. Cathepsin G inhibitor 1 (219372) was procured from Calbiochem, USA. Antibodies to cathepsin G; band 3 (polyclonal); protein kinase C (PKC) *β*II were from Abcam plc, UK (ab50845); Abnova, Taiwan (HOOOO6521-B01) Boehringer Mannheim, Germany (1471864), respectively. Enhanced chemiluminescence (ECL) plus (RPN 2132), protein G sepharose CL-4B (17-0780-01), and horse-radish peroxidase-(HRP-) conjugated secondary antibodies: anti-mouse (NA 931), anti-rabbit (NA 934), and anti-human (NA 933) IgGs were from GE Healthcare, UK. Polyvinylidene difluoride (PVDF) membrane (IPVH 00010) was from Millipore India Pvt. Ltd., India. Melon gel IgG purification kit (45206) was from Pierce Biotechnology, USA. *α*-Cyano-4-hydroxycinnamic acid (CHCA) (201344) was from Bruker Daltonics, Germany. Fluorescein-isothiocyanate-(FITC-) labelled anti-CD 15 (347423), anti-CD 45 (555482), antibodies were from BD Pharmingen, USA.

### 2.2. Biological Material

This study was undertaken after obtaining ethics clearance from the hospital ethics committee, and informed consent form was administered prior to sample collection. Peripheral blood (5 mL) was collected by venipuncture in ethylene-diamine-tetra-acetic-acid-(EDTA)-containing bulbs for separation of erythrocytes and non-EDTA containing bulbs for obtaining serum. Patients with confirmed diagnosis of CML-chronic phase, who received no prior treatment for the disease, were recruited for the study (*n* = 18). Patient information on age, sex, leukocyte count, and hemoglobin levels is given in Section  1 of the supplementary information. Healthy voluntary donors (N) who reported no health problems served as controls (*n* = 14).

### 2.3. Preparation of Erythrocyte Suspension

Erythrocytes were allowed to settle from the blood sample collected in EDTA bulbs. After removing the supernatant plasma, erythrocytes were washed three times in wash buffer (10 mM Tris pH 7.6, 150 mM NaCl) (1 : 40 v/v) separating them each time by centrifugation at 1500 rpm for 15 min at 4°C. The extent of neutrophil contamination in erythrocytes separated using this protocol was assessed by detection of CD 45- and CD 15-labelled cells by flow cytometry as detailed in Section  2 of supplementary information. The neutrophil contamination was less than 0.01% in the erythrocyte preparation.

### 2.4. Preparation of Membrane and Cytosol Fractions

The erythrocytes were lysed in equal volume of hypotonic solution (10 mM Tris pH 7.6, 1 mM EDTA, 20 *μ*g/mL PMSF) as described by Dodge et al. [14]. Membranes were recovered by centrifugation at 15,000 rpm for 15 min at 4°C in SS-34 rotor of Sorvall RC-5C centrifuge. The supernatant was preserved as the cytosolic fraction. The pellet containing erythrocyte membrane-skeletal fraction (referred to as membrane) was given three washes with wash buffer to remove the cytosolic proteins. Membrane as well as cytosolic fractions were aliquoted and preserved at −70°C until use. Protein was estimated using the modified Lowry's method [15].

### 2.5. Separation of Proteins by Sodium Dodecyl Sulfate-Polyacrylamide Gel Electrophoresis (SDS-PAGE), Western Blotting and Immunodetection of Band 3, Cathepsin G and PKC *β*II

Erythrocyte membrane proteins (60 *μ*g) were resolved on 10% SDS-polyacrylamide gels by electrophoresis and then transferred to PVDF membrane [16]. The blots were blocked with 5% nonfat dry milk in tris-buffered saline containing Tween-20 (TBST) (10 mM Tris, 150 mM NaCl, pH 7.4 containing Tween 20, 0.2%, v/v) at room temperature (RT) for 1 h and subsequently incubated overnight at 4°C with appropriate primary antibodies in 2.5% nonfat dry milk in TBST (anti-band 3 monoclonal, 1 : 5000; anti-band 3 polyclonal 1 : 1000; anti-cathepsin G 1 : 1000 dilution). After washing with TBST, the blots were incubated with anti-mouse HRP-conjugated secondary antibody with appropriate dilutions prepared in 2.5% non-fat dry milk in TBST for 1 h at RT. Following incubation, the blots were washed with TBST and were developed with ECL plus reagent, and the signal was recorded on X-ray films. PKC *β*II was detected similarly in the cytosolic fraction (120 *μ*g) using anti-PKC *β*II antibody (1 : 1000 dilution).

### 2.6. Detection of Erythrocyte Membrane Proteins Recognized by Serum IgG

IgG was separated from sera using Melon gel IgG purification kit as per manufacturer's instructions. Membrane proteins (60 *μ*g) were resolved on polyacrylamide gels in duplicate. The resolved proteins from one of the gels were transferred to PVDF membrane and probed with serum IgG and the other gel was silver stained to be used for identification of the antigenic proteins by mass spectrometry. Separation of proteins on SDS-PAGE, western blotting, and immunostaining was done using the protocol described above except for using 5 *μ*g of IgG separated from sera as primary antibody and HRP-labeled anti-human IgG as the secondary antibody. To detect proteins reacting with serum IgG, each membrane sample was tested with IgG separated from serum of the same individual.

### 2.7. Mass Spectrometric Identification of Erythrocyte Surface Antigen Bound by Serum IgG

The position of the band obtained on the autoradiograph after immunostaining was marked on the corresponding silver-stained gel, and the gel piece was processed for mass spectrometry as described earlier [17] except that the destained gel plug was reduced with 10 mM dithiothreitol and alkylated with 55 mM iodoacetamide before trypsinization. One *μ*L of the peptide digest extracted from a gel piece was premixed with equal volume of CHCA matrix and spotted on a matrix-assisted laser desorption ionization (MALDI) plate. Peptide mass fingerprint (PMF) data was acquired on the MALDI TOF-TOF (TOF: time of flight) mass spectrometer (Ultraflex II, Bruker Daltonics, Germany) in the reflector mode. The data was searched against Swiss-Prot database using MASCOT search engine with a peptide mass tolerance of 100 ppm.

### 2.8. Elucidation of the Role of Cathepsin G in Cleavage of Band 3

 To study the action of cathepsin G on erythrocyte membrane proteins, erythrocytes (10^8^ cells/mL) from six healthy volunteers were incubated at 50°C for 15 min in a water bath to simulate stress conditions. The cells were then treated with IgG-depleted (using protein G sepharose adsorption) and complement-inactivated (incubation at 50°C for 30 min) sera from healthy volunteers or CML patients in the presence or absence of pepstatin A (1 mg/mL) or cathepsin G inhibitor I (53 nM) at 37°C for 20 min. Thereafter, the erythrocytes were lysed, the membrane fractions, prepared, and protein, estimated as described earlier. The membrane protein fractions were resolved on SDS-PAGE, transferred on to PVDF membrane, and probed with anti-cathepsin G (diluted 1 : 1000) and anti-band 3 (monoclonal) antibodies as described earlier. The autographs with signals for band 3 were scanned using Epi-Chemi II gel documentation system (UVP, UK) and densitometric analysis of signal in each well was done using LabWorks software version 4.0.08.

## 3. Results and Discussion

### 3.1. Profile of Erythrocyte Membrane Band 3 from CML Patients and Controls

**Figure 1:**
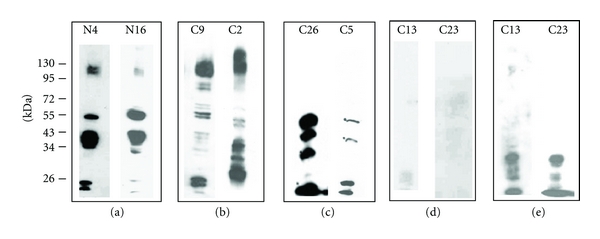
The profile of band 3 in the membranes of normal and CML erythrocytes. Western-blotted membrane proteins of erythrocytes (a–d) were stained with monoclonal anti-band 3 antibody. (a) The normal erythrocyte (N) shows the parent band 3 molecule at 95 kDa, and fragments at 55, 43, and below 26 kDa. (b–e) CML samples designated as “C” showed varied degree of fragmentation of band 3 as shown in (b) wherein parent band 3 is seen at 95 kDa and all fragments observed in normal are additionally cleaved (c) in which parent band 3 is not detected, and fragments detected in normal erythrocyte show greater intensity compared to the normal sample (d) where neither parent molecule nor the fragments are detected; (e) the fragments not detected in the representative CML sample in (d) are stained by polyclonal antibody to the whole band 3 molecule, thus indicating excessive cleavage of the N-terminal region which is detected by the monoclonal antibody.

Western blots of membranes prepared from normal and CML erythrocytes were stained with monoclonal anti-band 3 antibody. The band 3 profile obtained in representative normal samples is shown in Figure 1(a). It is seen that in addition to the parent band 3 molecule at approximately 95 kDa, fragments were detected at around 55, 43, and below 26 kDa in the normal erythrocyte samples. Cleavage of membrane protein band 3 is reported to occur in erythrocytes [18], and Kay et al. have shown that during normal erythrocyte aging, limited proteolysis of band 3 generates peptides of 60, 42, and 18–26 kDa [5], which is similar to our observation. This fragmentation is attributed to activation of membrane-bound proteases [19]. Caspase 3, which is reported to be active in aged erythrocytes [20], cleaves the N-terminal region of band 3 to generate two fragments of 18 and 21 kDa [21] and can explain the detection of a faint doublet observed below 26 kDa in Figure 1(a).

 Normal-like pattern of band 3 was observed only in 2/18 (11%) CML samples. The majority of CML samples (89%) showed extensive fragmentation of band 3. Three different band 3 profiles were seen for CML samples (Figures 1(b), 1(c), and 1(d)). The membrane fractions of erythrocytes from 13/18 CML samples showed either additional cleavage of the fragments observed in normal erythrocytes (Figure 1(b)) or greater intensity of the fragments as compared to the normal with near undetectable parent band 3 (Figure 1(c)). Three out of eighteen CML samples did not stain with the monoclonal antibody as shown in the representative samples in Figure 1(d). However, the fragments were detected when the blot was stained with polyclonal anti-band 3 antibody against the whole band 3 protein (Figure 1(e)), which indicates cleavage of N-terminal domain of the molecule, recognized by the monoclonal antibody.

Fragmentation of band 3 bears a physiological significance as distinct fragments are senescent cell antigens [5, 6], which elicit immune recognition and phagocytosis of aged erythrocytes. It was thus appropriate to investigate if the fragment/s of band 3 detected in the membrane of CML erythrocytes were recognized by serum IgG.

### 3.2. Antigens Recognized by Serum IgG in the Membranes of Erythrocytes from CML Patients and Normal Individuals

**Figure 2:**
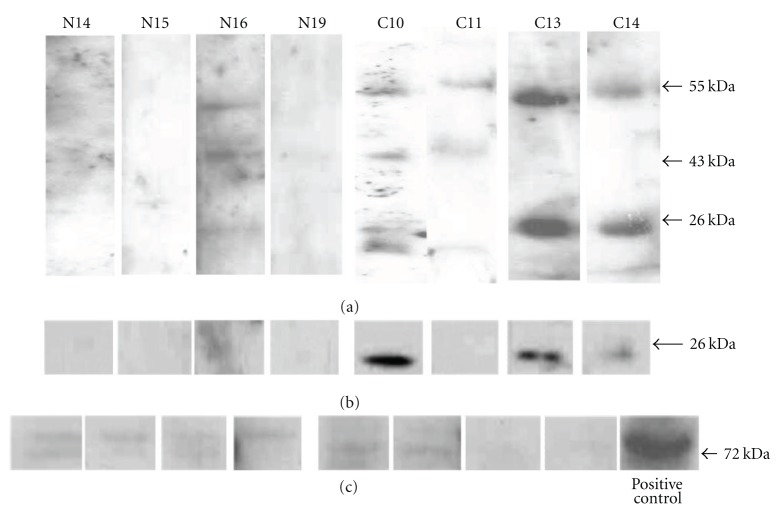
Detection of Antigens on erythrocyte membrane which are recognized by serum IgG. (a) autographs of western blots of membranes of erythrocytes from CML patients designated C, and controls (N) stained with IgG separated from sera of the respective individual have been shown. A 43 kDa fragment is stained in normal and a larger number of CML while fragments at 55 and 26 kDa are stained mainly in CML erythrocytes. The antigens are identified as fragment of band 3 (43 kDa), myeloperoxidase (55 kDa), and cathepsin G (26 kDa) by mass spectrometry (details in Table 1). (b) The identity of cathepsin G was validated by immunostaining of western blots of erythrocyte membranes with cathepsin G-specific antibody. CML samples show higher expression of cathepsin G. (c) Immunostaining of blotted proteins from cytosol of normal and CML erythrocytes with PKC *β*II antibody shows a low-intensity band in both. PKC *β*II is expressed in neutrophils but not in erythrocytes. Insignificant detection of PKC *β*II in both normal and CML samples rules out the detection of cathepsin G due to contamination of neutrophils in the erythrocyte preparation. Rat brain lysate is the positive control.

To determine whether serum IgG recognized any of the band 3 fragments, the western blots of membrane proteins from normal and CML erythrocytes were probed with IgG purified from the respective sera. Bands corresponding to those detected by serum IgG were excised from a replicate silver-stained gel and analyzed by mass spectrometry. The antibodies recognized an immunogen at 43 kDa in 3/8 (38%) normal and 6/9 (68%) CML samples (representatives in Figure 2(a), lanes N16, C10, and C11), which was identified as a fragment of band 3 by mass spectrometry (Table 1). Thus CML erythrocytes showed greater fragmentation of band 3 (Figures 1(b), 1(c), and 1(e)) as well as wider occurrence of immunogenic band 3 fragments (Figure 2(a)) and thereby eryptotic phenotype. This is in keeping with the detection of a 45 kDa immunogenic fragment of band 3 reported by Santos-Silva et al. [2] in postmyocardial infarction patients and controls but differs from the report on the recognition of a 62 kDa band 3 fragment by IgG eluted from aged erythrocytes by Kay [6].

Additionally, proteins at 55 kDa and 26 kDa were recognized by serum IgG in 8/9 samples from CML patients (Figure 2(a)) and only 3/6 healthy volunteers (Figure 2(a), representative in lane N16). The molecules at 55 kDa and 26 kDa, which were detected by serum IgG in the majority of CML samples, were identified as myeloperoxidase and cathepsin G, respectively, by mass spectrometry (Table 1). Both proteins are abundant in azurophilic granules of neutrophils and are not expressed in erythrocytes.

To confirm the presence of cathepsin G in the erythrocyte membranes, the western-blotted membrane proteins from normal and CML samples were probed with anti-cathepsin G antibody. Figure 2(b) shows that cathepsin G was detected mainly in CML samples. The possibility of neutrophil contamination contributing to the increased cathepsin G levels in CML erythrocyte membrane preparations was ruled out by demonstrating nondetection of PKC *β*II (Figure 2(c)), which is present in neutrophils [22] but not erythrocytes [23]. The detection of cathepsin G, which is a protease, in erythrocyte membranes with excessively fragmented band 3 suggested a possible role of the former in the fragmentation of the later.

### 3.3. Adsorption of Serum Cathepsin G to Erythrocyte Membranes and Cleavage of Band 3

Detection of the neutrophilic cathepsin G on erythrocyte membrane required investigations on its source. Stimulated neutrophils are known to release elastase and cathepsin G in the surrounding medium [24] and stressed erythrocytes adsorb proteins from body fluids [25]. In this study, stress condition was simulated by incubation of normal erythrocytes at 50°C for 30 min, and the stressed erythrocytes were further exposed to IgG-depleted/complement-inactivated sera from CML patients and normal controls.

**Figure 3:**
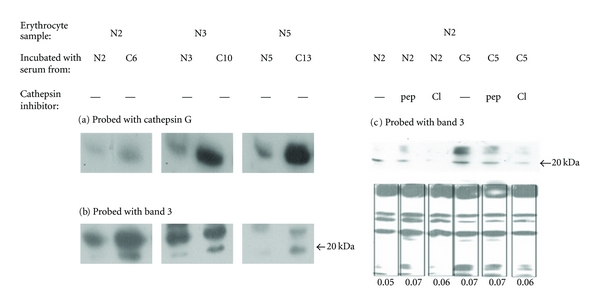
*In vitro* studies to delineate the role of cathepsin G in the proteolysis of band 3. Heat-stressed normal (N) erythrocytes were incubated with IgG-depleted/complement-inactivated sera from the same individual and also with sera from CML patients designated C (N2 with C6, N3 with C10 and N5 with C13) at 37°C for 20 min. Western blots prepared from the lysates of these erythrocytes were stained with cathepsin G and band 3 antibodies. (a) Cathepsin G levels are higher in the membrane of erythrocytes incubated with CML sera as compared to normal sera. (b) Staining of the same blot with anti-band 3 antibody shows that a 20 kDa fragment of band 3 is seen in cells incubated with CML sera. (c) The generation of 20 kDa fragment is diminished upon incubation with pepstatin A (pep) and is nearly inhibited by cathepsin G inhibitor 1 (CI). Densitometric readings of each lane of the band 3 autograph corresponding to the lanes in (c) demonstrate equal loading.

Erythrocytes from healthy volunteers incubated with sera from CML patients showed higher levels of membrane cathepsin G as compared to those incubated with sera from controls as shown in Figure 3(a). Cathepsin G being a serine protease, the natural sequel to this observation was to investigate if cathepsin G had a role in the cleavage of band 3 in CML erythrocytes. The same set of samples was probed with anti-band 3 antibody. Figure 3(b) shows that a band 3 fragment at approximately 20 kDa is detected in membranes of erythrocytes incubated with sera of CML patients with a band of lower intensity detected only in 1/3 samples incubated with normal serum (N3 in Figure 3(b)). Incubation with sera from normal or CML patients in the presence of pepstatin A, which can inhibit cathepsin E known to be expressed in erythrocytes [26], caused moderate decrease in the generation of the 20 kDa fragment of band 3 (Figure 3(c)). Inhibition of generation of a 55 kDa fragment of band 3 by pepstatin A is reported in Ca2+-enriched erythrocytes [27]. In our study, a near complete inhibition of formation of the 20 kDa fragment was observed upon incubation of erythrocyte with either normal or CML sera in the presence of cathepsin G inhibitor 1 (Figure 3(c)) which demonstrated the involvement of serum cathepsin G in the cleavage of band 3 *in vitro*. The densitometric values for the band 3 signals for each lane as shown below Figure 3(c) indicated equal detection of total band 3 in the presence and absence of pepstatin A and cathepsin G inhibitor 1. This confirmed that the inhibition observed was not an artifact of unequal loading.

Although neutrophilic elastase has been shown to cleave erythrocyte band 3 [7], ours is the first report on involvement of cathepsin G in the proteolysis of band 3. In contrary to our observation, Bykowska et al. [8] have shown that band 3 is partially digested by elastase but is resistant to cleavage by cathepsin G. The difference could be due to the use of pure cathepsin in their study against serum used in our investigation, which contains several molecules other than cathepsin G. It can be speculated that factors other than cathepsin G in serum could cause exposure of a cryptic site on band 3, which is proteolyzed by cathepsin G. It is also possible that cleavage of glycophorin by cathepsin G removes steric “shielding” of band 3 by the adjacent glycophorins [28], thereby allowing cleavage of band 3 by another protease, such as elastase.

## 4. Conclusion

Thus, co-occurrence of extensively cleaved band 3 and adherent cathepsin G on the membranes of CML erythrocytes, along with the *in vitro* demonstration of the role of cathepsin G in proteolysis of band 3, indicates involvement of cathepsin G in band 3 cleavage *in vivo*. Moreover, the excessive cleavage of band 3 in CML correlates with greater detection of immunogenic fragments of band 3, indicative of eryptotic phenotype. Thus, cathepsin G-mediated proteolysis of band 3 could exemplify a mechanism for the generation of eryptotic phenotype in CML.

## Figures and Tables

**Table 1:** Mass spectrometric identification of erythrocyte membrane antigens recognized by serum IgG. Trypsin digests of protein spots in gels of erythrocyte membranes, at a position corresponding to signals for immunogens recognized by serum IgG in the western blot of a replicate gel, were subjected to mass spectrometry. Peptide mass finger print (PMF) data was acquired on the MALDI TOF-TOF Protein analyzer (Ultraflex II, Bruker Daltonics) in the reflector mode. The data was searched against SwissProt database using MASCOT search engine with a peptide mass tolerance of 100 ppm.

Molecular weight in SDS-PAGE	Sample	Accession no.	pI	Molecular weight in kDa	Score	Sequence coverage	Peptides matched (submitted)	Protein identity
55 kDa	CML 10	PERM_H	9.19	84.7	118	14%	11 (14)	Myeloperoxidase
	CML 11	PERM_H	9.19	84.7	94	11%	8 (10)	
	CML 13	PERM_H	9.19	84.7	61	8%	6 (15)	
	CML 14	PERM_H	9.19	84.7	70	8%	7 (13)	

43 kDa	N6	B3AT_H	5.08	102	74	11%	7 (22)	Band 3 anion transport protein
	N11	B3AT_H	5.08	102	66	19%	14 (52)	
	CML 11	B3AT_H	5.08	102	50	17%	10 (48)	

26 kDa	CML 12	CATG_H	11.19	29.1	64	17%	5 (11)	Cathepsin G
	CML 13	CATG_H	11.19	29.1	71	26%	6 (17)	
	CML 14	CATG_H	11.19	29.1	64	25%	6 (29)	
	CML 16	CATG_H	11.19	29.1	70	22%	7 (25)	

## References

[1] Segel GB, Halterman MW, Lichtman MA (2011). The paradox of the neutrophil’s role in tissue injury. *Journal of Leukocyte Biology*.

[2] Santos-Silva A, Molnar Bayer Castro E, Almeida Teixeira N, Carvalho Guerra F, Quintanilha A (1995). Altered erythrocyte membrane band 3 profile as a marker in patients at risk for cardiovascular disease. *Atherosclerosis*.

[3] Santos-Silva A, Rebelo I, Castro E (2002). Erythrocyte damage and leukocyte activation in ischemic stroke. *Clinica Chimica Acta*.

[4] Belo L, Rebelo I, Castro EMB (2002). Band 3 as a marker of erythrocyte changes in pregnancy. *European Journal of Haematology*.

[5] Kay MMB, Goodman SR, Sorensen K (1983). Senescent cell antigen is immunologically related to band 3. *Proceedings of the National Academy of Sciences of the United States of America*.

[6] Kay MMB (1984). Localization of senescent cell antigen on band 3. *Proceedings of the National Academy of Sciences of the United States of America*.

[7] Santos-Silva A, Castro EMB, Teixeira NA, Guerra FC, Quintanilha A (1998). Erythrocyte membrane band 3 profile imposed by cellular aging, by activated neutrophils and by neutrophilic elastase. *Clinica Chimica Acta*.

[8] Bykowska K, Duk M, Kusnierz-Alejska G, Kopec M, Lisowska E (1993). Degradation of human erythrocytre surface components by human neutrophil elastase and cathepsin G: preferential digestion of glycophorins. *British Journal of Haematology*.

[9] Bykowska K, Duk M, Kusnierz-Alejska G (1997). Degradation of glycophorin A of human erythrocytes in patients with myelo- or lymphoproliferative disorders: possible role of neutrophil proteases. *British Journal of Haematology*.

[10] Kumar A, Gupta CM (1983). Red cell membrane abnormalities in chronic myeloid leukaemia. *Nature*.

[11] Kundu M, Basu J, Chakrabarti P (1990). Chronic myelogenous leukemia: alterations in red cell membrane band 3 and increased IgG binding. *Indian Journal of Biochemistry and Biophysics*.

[12] Kay MMB, Flowers N, Goodman J, Bosman G (1989). Alteration in membrane protein band 3 associated with accelerated erythrocyte aging. *Proceedings of the National Academy of Sciences of the United States of America*.

[13] Kubes P, Smith R, Grisham MD, Granger DN (1993). Neutrophil-mediated proteolysis. Differential roles for cathepsin G and elastase. *Inflammation*.

[14] Dodge JT, Mitchell C, Hanahan DJ (1963). The preparation and chemical characteristics of hemoglobin-free ghosts of human erythrocytes. *Archives of Biochemistry and Biophysics*.

[15] Peterson GL (1977). A simplification of the protein assay method of Lowry et al. Which is more generally applicable. *Analytical Biochemistry*.

[16] Towbin H, Staehelin T, Gordon J (1979). Electrophoretic transfer of proteins from polyacrylamide gels to nitrocellulose sheets: procedure and some applications. *Proceedings of the National Academy of Sciences of the United States of America*.

[17] Govekar RB, D’Cruz AK, Pathak KA (2009). Proteomic profiling of cancer of the gingivo-buccal complex: identification of new differentially expressed markers. *Proteomics—Clinical Applications*.

[18] Arese P, Turrini F, Schwarzer E (2005). Band 3/complement-mediated recognition and removal of normally senescent and pathological human erythrocytes. *Cellular Physiology and Biochemistry*.

[19] Tarone G, Hamasaki N, Fukuda M, Marchesi VT (1979). Proteolytic degradation of human erythrocyte band 3 by membrane-associated protease activity. *Journal of Membrane Biology*.

[20] Mandal D, Moitra PK, Saha S, Basu J (2002). Caspase 3 regulates phosphatidylserine externalization and phagocytosis of oxidatively stressed erythrocytes. *FEBS Letters*.

[21] Mandal D, Baudin-Creuza V, Bhattacharyya A (2003). Caspase 3-mediated proteolysis of the N-terminal cytoplasmic domain of the human erythroid anion exchanger 1 (band 3). *Journal of Biological Chemistry*.

[22] Balasubramanian N, Advani SH, Zingde SM (1998). Protein kinase C isoforms in normal and chronic myeloid leukemic neutrophils. Distinct signal for PKC *α* by immunodetection on PVDF membrane, decreased expression of PKC *α* and increased expression of PKC *δ* in leukemic neutrophils. *Leukemia Research*.

[23] Govekar RB, Zingde SM (2001). Protein kinase C isoforms in human erythrocytes. *Annals of Hematology*.

[24] Bangalore N, Travis J (1994). Comparison of properties of membrane bound versus soluble forms of human leukocytic elastase and cathepsin G. *Biological Chemistry Hoppe-Seyler*.

[25] Smirnov II, Levin VN, Zdiumaeva NP (2004). Protein adsorption on erythrocytic membranes and its effect on erythrocyte rheology in athletes during competition exercise. *Fiziologiia cheloveka*.

[26] Takeda-Ezaki M, Yamamoto K (1993). Isolation and biochemical characterization of procathepsin E from human erythrocyte membranes. *Archives of Biochemistry and Biophysics*.

[27] Lorand L, Bjerrum OJ, Hawkins M (1983). Degradation of transmembrane proteins in Ca^2+^ -enriched human erythrocytes. An immunochemical study. *Journal of Biological Chemistry*.

[28] Kay MMB, Goodman JR (1984). IgG antibodies do not bind to band 3 in intact erythrocytes; enzymatic treatment of cells is required for IgG binding. *Biomedica Biochimica Acta*.

